# Editorial: Noncoding RNAs in neurodegenerative disorders: from current insights and future directions to translational modeling and therapeutic approaches

**DOI:** 10.3389/fnins.2024.1497673

**Published:** 2024-10-29

**Authors:** Yujing Li, Akshay Bhinge, Satoshi Inoue, Gonçalo Garcia

**Affiliations:** ^1^Department of Human Genetics, Emory University School of Medicine, Atlanta, GA, United States; ^2^College of Medicine and Health, University of Exeter, Exeter, United Kingdom; ^3^Department of Systems Aging Science and Medicine, Tokyo Metropolitan Institute of Gerontology, Tokyo, Japan; ^4^Research Institute for Medicines (iMed.ULisboa), Faculty of Pharmacy, Universidade de Lisboa, Lisbon, Portugal; ^5^Department of Pharmaceutical Sciences and Medicines, Faculty of Pharmacy, Universidade de Lisboa, Lisbon, Portugal

**Keywords:** non-coding RNA, neurodegenerative disorders (NDs), Alzheimer's disease (AD), Parkinson's disease (PD), amyotrophic lateral sclerosis (ALS), miRNA, piRNA, lncRNA

Neurodegenerative disorders (NDs), such as Alzheimer's disease (AD), Parkinson's disease (PD), and amyotrophic lateral sclerosis (ALS), remain major challenges in medicine, characterized by the gradual loss of neuronal structure and function (Mo, 2023; Canoy et al., 2024). Many research studies have shed light on the crucial roles of noncoding RNAs (ncRNAs) in the pathogenesis of these diseases, offering new avenues for diagnosis and therapy (Garcia et al., 2021; Canoy et al., 2024). This Research Topic focuses on the involvement of various ncRNAs, including piRNAs, microRNAs (miRNAs), long noncoding RNAs (lncRNAs), circular RNAs (circRNAs) and competing endogenous RNAs (ceRNAs), in the NDs, as well as exploring future directions for translating these findings into clinical therapies. The studies presented in this Research Topic provide valuable insights into the molecular mechanisms governed by the ncRNAs and their potential to serve as both biomarkers and therapeutic targets.

## Role of piRNA biogenesis in neurodegeneration

In their Research Topic, Sato et al., delves into piRNAs, a class of small ncRNAs involved in silencing transposable elements and maintaining genomic stability. While piRNAs are well-studied in the context of germline cells, their role in neurons is less understood. The authors explored how impaired piRNA biogenesis could contribute to neurodegenerative processes through genomic instability, particularly in long-lived neurons. They suggest that defects in piRNA pathways could render neurons more susceptible to DNA damage, promoting neurodegeneration. Given their regulatory role, piRNAs could be novel targets for therapeutic intervention aimed at stabilizing the neuronal genome during the neurodegeneration.

## Unveiling ceRNA networks in astrocytic response to lipotoxicity

Astrocytes play critical roles in maintaining the central nervous system (CNS) homeostasis, and disruptions in their function are closely linked to neurodegeneration. Gil-Jaramillo et al., investigates how ceRNA networks in astrocytes respond to lipotoxic stress, a condition closely associated with metabolic dysfunction and neurodegeneration. The study highlights the complex interplay between ncRNAs and ceRNAs, such as lncRNAs and circRNAs, in regulating lipid-induced damage. Alterations in these networks were reported to lead to astrocytic dysfunction, contributing to neuronal injury. The research underscores the therapeutic potential of targeting ceRNA networks to restore astrocyte function and mitigate neurodegeneration.

## An overview about the miR-124's roles and traumatic brain injury

Traumatic brain injury (TBI) is a known risk factor for developing NDs later in life. Wu et al., provides a comprehensive review of miR-124, a brain-enriched miRNA implicated in neuroinflammation, synaptic plasticity, and neuronal survival following TBI. The authors explore the dual function of miR-124, which can either protect against or contribute to neurodegeneration depending on the context of injury and the involved molecular pathways. The authors also discuss how therapeutic modulation of miR-124 could potentially reduce neuroinflammation and promote neurodegeneration, offering a potential strategy to reduce the long-term risk of developing NDs such as AD and PD for the individuals with a history of TBI.

## miRNAs and PD: a genome-wide approach

PD, characterized by the progressive loss of dopaminergic neurons, is influenced by both genetic and environmental factors. Shi et al., adopts a novel genome-wide approach to identify miRNAs with causal links to PD using Mendelian randomization. The study identifies several miRNAs that regulate PD-associated genes, providing new insights into the molecular pathways underlying the disease and emphasizing the potential of miRNAs as both biomarkers for early diagnosis and targets for disease-modifying therapies in PD.

## Translational modeling and future directions

Together, these studies discussed in this Research Topic underscore the intricate regulatory roles of different classes of ncRNAs in the NDs and point to multiple promising avenues for future research in molecular mechanisms of the pathogenesis and the development of therapeutic strategies:

1. Therapeutic modulation of ncRNAs: the ncRNA-based therapy offers promising new avenues for the clinical therapy for the NDs (Chen et al., 2017; Wang et al., 2022). Therapeutic strategies designed to restore the balance of miRNAs, piRNAs, lncRNAs, circRNAs, and ceRNAs could provide broad neuroprotective effects, targeting multiple molecular pathways simultaneously (Chen et al., 2017). While RNA interference (RNAi) and antisense oligonucleotide (ASO) therapies are being actively explored as innovative interventions, they remain at the early stages in terms of clinical applications, with significant opportunities for refinement and optimization (Wang et al., 2022).
2. ncRNAs as biomarkers: the accessibility of ncRNAs in body fluids positions them as ideal candidates for non-invasive biomarkers. By monitoring specific ncRNA signatures over time, clinicians could easily track disease progression and therapeutic responses with greater precision (Chen et al., 2017).
3. Integrative omics and computational modeling: advanced bioinformatics and integrative omics approaches, such as RNA sequencing and proteomics, could provide critical tools for deciphering the complex regulatory networks involving ncRNAs and their targeting arrays, as well as their associations with disease phenotypes (Wang et al., 2022). This will help identify the best therapeutic targets and refine our understanding of ncRNA function in neurodegeneration.

Overall, the studies described in this Research Topic offer crucial insights into the role of ncRNAs in NDs, illustrating their great potential values serving as not only biomarkers but also therapeutic targets. Future research aimed at understanding the molecular mechanisms of ncRNAs, combined with advances in RNA-based therapies, holds great promise for improving outcomes in NDs. As we move toward a future where ncRNA modulation becomes a part of the therapeutic landscape, the potential for meaningful clinical advances in treating these devastating diseases becomes increasingly attainable.
